# Activation of PKCzeta and PKMzeta in the Nucleus Accumbens Core Is Necessary for the Retrieval, Consolidation and Reconsolidation of Drug Memory

**DOI:** 10.1371/journal.pone.0030502

**Published:** 2012-02-10

**Authors:** Jose A. Crespo, Petra Stöckl, Florian Ueberall, Marcel Jenny, Alois Saria, Gerald Zernig

**Affiliations:** 1 Experimental Psychiatry Unit, Department of General Psychiatry and Social Psychiatry, Innsbruck Medical University, Innsbruck, Austria; 2 Innsbruck Biocentre, Division of Medical Biochemistry, Innsbruck Medical University, Innsbruck, Austria; University of Chicago, United States of America

## Abstract

One of the greatest challenges in the treatment of substance dependence is to reverse the control that drug-associated stimuli have gained over the addict's behavior, as these drug-associated memories increase the risk of relapse even after long periods of abstinence. We report here that inhibition of the atypical protein kinase C isoform PKCzeta and its constitutively active isoform PKMzeta with the pseudosubstrate inhibitor ZIP administered locally into the nucleus accumbens core reversibly inhibited the retrieval of drug-associated memory and drug (remifentanil) seeking, whereas a scrambled ZIP peptide or staurosporine, an effective inhibitor of c/nPKC-, CaMKII-, and PKA kinases that does not affect PKCzeta/PKMzeta activity, was without effect on these memory processes. Acquisition or extinction of drug-associated memory remained unaffected by PKCzeta- and PKMzeta inhibition.

## Introduction

Drug dependence has remained a formidable therapeutic challenge because, among others, the underlying maladaptive behavior is remarkably resistant to change, even when such a change is the focus of psychotherapeutic interventions [1]. Recent animal experimental findings, however, indicate that drug-associated memories may be amenable to genetic/pharmacologic manipulation during the memory reconsolidation phase [2], [3]: Cocaine-conditioned place preference could be inhibited by bilateral local administration into the nucleus accumbens core (AcbC) of inhibitors of mitogen-activated protein kinases (MEKs) [4] or antisense oligonucleotides of the immediate early gene zif268 (egr1) [5], while acquisition of a new operant response could be blocked by zif268 knockdown in the basolateral amygdala (BLA) [5], [6], [7] but not the AcbC (see Discussion).

We could recently demonstrate that activation of both muscarinic and nicotinic acetylcholine receptors located in the AcbC is necessary for the acquisition of rat runway behavior conditioning by drugs of abuse [8] but not food [9] (but see [10], [11], confirming previous findings obtained in a lever-press-based self-administration paradigm [12]. Muscarinic acetylcholine receptors (mAChRs) are known to activate conventional, novel, and atypical isotypes of the protein kinase C (PKC) family [13]. Activation of atypical PKCs (aPKCs) was found to be required for the firing of Acb medium spiny neurons [14], the activation of which is necessary for operant behavior under a second order schedule of responding [15]. We therefore investigated the expression, phosphorylation and activity of PKC isoforms, in particular PKMzeta, in the AcbC during the consolidation, storage, retrieval, and reconsolidation of associative memories involved in drug seeking. To that end, we employed the rat runway paradigm, because of its considerable face validity for the human situation and because it is well suited to quantify the control that drug-associated contextual stimuli exert over an individual's behavior [16], moved to Discussion: although the most differentiating behavioral analysts [17] emphasize that the rat runway paradigm, like the conditioned place preference (CPP) procedure and other maze paradigms, is not able to distinguish between Pavlovian (i.e., respondent) approach (insensitive to changes in action-outcome contingencies and thus not truly goal-directed) and operant behavior (sensitive to changes in action-outcome contingencies). We shall therefore use the term “(conditioned) approach to drug” to describe the rats' behaviour because this term reflects the minimum theoretical consensus that can be obtained.

As a prototypical drug of abuse, we chose to test remifentanil instead of cocaine (arguably the most popular experimental drug of abuse), because the mu opioid agonist remifentanil has a much less complex signal transduction pathway than cocaine while displaying the same pharmacokinetic properties as cocaine: fast distribution into the brain, fast elimination from deep brain structures, and fast esteratic degradation in the blood [18], [19]. In addition, self-administration of remifentanil is proportional over a much larger unit dose range than cocaine, most likely because self-administration of remifentanil is much less limited by the compound's concurrent aversive properties than that of cocaine [16].

## Materials and Methods

### Subjects and animal care

Male Sprague Dawley rats were obtained from the Research Institute of Laboratory Animal Breeding (Himberg, Austria) weighing 250–300 g on receipt. Before surgery, all animals were housed in groups of six at a constant room temperature of 24°C and had free access to tap water and food. All rats were tested during the light phase of a 12-hr light-dark cycle (lights on at 0700 h). The animals used in this study were cared for in accordance with the guidelines of the National Institutes of Health Animal Care and Use Program and the NIDA-IRP Animal Program, which is fully accredited by the Association for Assessment and Accreditation of Laboratory Animal Care International. Furthermore, the present experiments were approved by the national Animal Experiment Ethics Committee. Morphine was obtained from the Innsbruck University Hospital Pharmacy, all other chemicals were obtained from Sigma-Aldrich (Vienna, Austria) unless indicated otherwise. Doses and concentrations refer to pure base.

### Implantation of intravenous catheters and intra-accumbens infusion cannulae

Male Sprague Dawley rats were implanted under isoflurane (2–4%; Abbott, Vienna) anesthesia with guide cannulae 48 h before the actual experiments and previously with self-made jugular vein catheters [8] with the following dimensions: 0.6 mm inner diameter (ID)×1.2 mm outer diameter (OD)×0.3 mm silicone tubing thickness. On test day, infusion cannulae were advanced to tip coordinates of AP+1.6 mm relative to bregma, ML - 1.6 mm, and DV 8.2 mm [20]. Only experiments in which the cannula tip location was confirmed by visual inspection of post-mortem brain slices to be within the AcbC limits as defined in the Paxinos and Watson atlas were included in the study.

### Runway behavior

In the operant runway procedure [21], [22] [9], [23], the time that an animal needs to obtain a stimulus, the “runtime”, is commonly thought to be inversely proportional to the apparent [16] reinforcing strength of that stimulus Using the runway procedure, we could reliably demonstrate acquisition of drug seeking for opioids and psychostimulants within only five consecutive trials in completely drug- and experiment-naive rats [8], [9], [18], [22]. In the present study, two days after the implantation of the intravenous (i.v.) catheters and the guide cannulae, completely drug- and experiment-naïve rats were given the opportunity to run for access to an i.v. injection of 0.032 mg/kg remifentanil (www.glaxosmithkline.com) for four consecutive trials (“runs”; intertrial interval, 40 min). This remifentanil dose was chosen because it was the highest dose that proved to be a reliable positive reinforcer in previously published runway experiments from our group (see Figure 3 of [22]) and was shown to to result in a robust in vivo microdialysis neurotransmitter release signal [8]. The inter-trial interval of 40 min was chosen to allow elimination of >90% of the drug from the AcbC between runs (see Figure 1 of [8] to avoid direct pharmacological effects of remifentanil (e.g., sedation). Runs were started by opening a sliding door separating a start area from the main alley (length, 1 m) of the runway and by indicating availability of i.v. remifentanil with a white cue light in the goal area. The click of a photobeam and the blinking of the cue light indicated the successful completion of the response for the run-contingent remifentanil infusion. Offline behavioral analysis of tapes by a treatment-blinded experimenter [22] had shown that when a positive reinforcer is made available in the goal area, the rats immediately leave the start area, i.e., commit to approaching the reinforcer-associated goal area immediately, and approach the goal area without engaging in alternative behavior. In contrast, both an increase in the latency to leave the start area and an increase in alternative behavior in the runway alley had been consistently observed with saline as compared to i.v. drugs of abuse. Consequently, three behavioral components were recorded, ie (1) the start area time, defined as the time elapsed between the placement of the animal within the start area and the exit of the whole animal from the area once the sliding door had been opened by the researcher; (2) the alley time as the time elapsed between the animal's exit of the start area and the entry of the whole animal into the goal area, and (3) the goal time as the duration of the animal's stay in the goal area with the sole administration of a CS+ (up to 15 seconds) until it retreated back leaving the area completely. For the animals that received an US+ (i.e., remifentanil) just after a complete entry, such a time was not measured due to the direct effects of the drug on locomotion. Runway dimensions and experimental details have been published previously [22], [24], [25]. Details of the experimental design and timeline could be found in Table 1.

**Figure 1:**
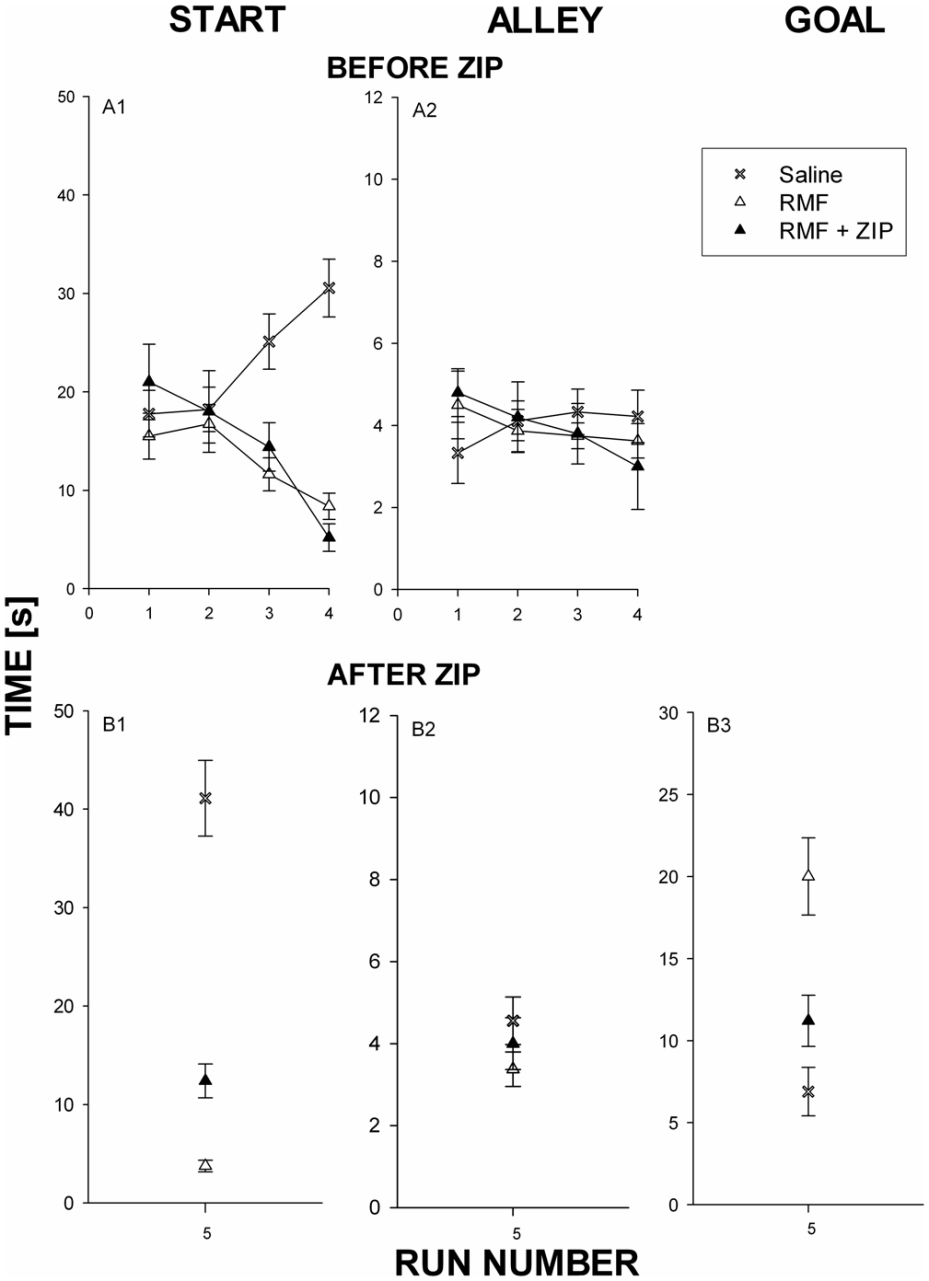
Local intra-AcbC inhibition of PKC/Mzeta the retrieval of conditioned remifentanil approach-associated memories. (A) Runway behavior before ZIP administration, runs #1-4: Drug-naïve male Sprague Dawley rats were given the opportunity to traverse a runway alley to obtain 0.032 mg/kg i.v. RMF (contingent RMF administration, i.e., RMF self-administration, 40 min inter-run interval; RMF, N = 13) or saline (SAL, N = 9) paired with a light stimulus (conditioned stimulus, CS; blinking at 2 Hz for 20 s)[8] (B) Runway behavior after ZIP administration, run #5: In order to determine the goal time in absence of the drug with its confounding sedative effects, RMF was not administered in this last run. The PKCzeta- und PKMzeta-pseudosubstrate inhibitor ZIP (0.5microliter of 1.5 mM) was administered locally into the nucleus accumbens core (30 min before run #5) of five animals after the first four RMF runs (RMF+ZIP). Column 1 shows start time (i.e., latency to leave the start area), column 2 alley time (i.e., time needed to traverse the runway alley), and column 3 goal time (i.e., time spent in the goal area). In order to determine the effect of noncontingent RMF (i.e., the acute pharmacological effects of RMF) on PKCzeta- and PKMzeta activation (see Fig. 3), another group of animals passively received i.v. RMF within the confines of the runway (noncontingent RMF administration, N = 10). Their runway behavior was not recorded and is therefore not shown here. The statistical analysis gave the following results: Panel A1, 2W-RM-ANOVA, Interaction [F(6,19)] = 7,875 P<0.0001. Group [F(2,19)] = 16,44 P<0.0001, post-hoc (Bonferroni): ZIP vs Saline (run3: p<0.05, run4: p<0.001), RMF vs Saline(run3: p<0.001, run4:p<0.001). Panel B1: 1W-ANOVA F(2,19) = 52.90 p<0.0001, post-hoc (Bonferroni): RMF+ZIP vs Saline (p<0.001), RMF+ZIP vs RMF (ns), RMF vs Saline (p<0.001)). Panel B3, 1W-ANOVA F(2,19) = 13,66 p<0,0002, post-hoc (Bonferroni): RMF+ZIP vs Saline (ns), RMF+ZIP vs RMF (p<0.05), RMF vs Saline (p<0.001)).

**Table 1:** Details of the behavioral experimental design and timeline.

Experiment 1		acquisition	admin	test
	run	1–4	(30′)	5
RMF (*n* = 13)		rmf	veh	x
RMF+ZIP (*n* = 5)		rmf	ZIP	x
Saline (*n* = 9)		sal	veh	x

Experiment 2		acquisition (day 1)				test (day 2)	reminder (day 3)	reacq. (day 4)	test
	run	1	2	3	4	1–3	1–2	1–5	6
RMF+ZIP (*n* = 7)		↓ZIP	rmf	rmf	↓ZIP	x	rmf	rmf	x
RMF+ZIP scr (*n* = 8)		↓ZIPs	rmf	rmf	↓ZIPs	x	rmf	rmf	x
RMF+Stauro (*n* = 7)		↓Stau	rmf	rmf	↓Stau	x	rmf	rmf	x

Experiment 3						
		acquisition (day 1)	ZIP admin (day 2)	retrieval	reinst. (day 4)	reacq. (day 5)
	run	1–14	(30′)	1–8	1–7	1–5
RMF+ZIP (*n* = 9)		rmf	↓	x	rmf (1)	rmf
RMF+ZIP scr (*n* = 10)		rmf	↓	x	rmf (1)	rmf
RMF+ZIP control (n = 7)		rmf	↓		rmf (1)	rmf

rmf: remifentanil, veh: vehicle, sal: saline, admin: administration, reacq: reaquisition, reinst: reinstatement, stau: staurosporine, zips: zip scramble, x: trials without drug reinforcement, ↓: intraaccumbens injection, rmf(1): self-administration of remifentanil only after completion of the first run.

### Brain tissue lysate preparation and subcellular fractionation

Rats were sacrificed at the end of the experiment, their brains were immediately recovered, generously washed in ice-cold saline, and were dissected immediately on ice. The tissue was immediately homogenized with a mechanical tissue disrupter in hypotonic lysis buffer (20 mM Tris-HCl, pH 7.5, 2 mM MgCl_2_, 10 mM KCl, 10 mM ß-glycerophosphate, 5 mM Na_4_P_2_O_7_×10 H_2_O, 2 mM EDTA, 2 mM EGTA, protease inhibitor mix HP from www.serva.de), was incubated on ice for 30 min and was centrifuged at 250× g to get rid of debris. The lysate was subsequently centrifuged at 100,000× g for 1 h, and the supernatant was stored as “cytosolic fraction” at −70°C. The pellet was resuspended in hypotonic lysis buffer+2% Triton X-100 with repeated vortexing for 1 hour on ice, and was stored as “membrane fraction” at −70°C.

### Immunoblotting

Subcellular fractions as well as non-fractionated lysates (homogenized in lysis buffer with 50 mM Tris, 50 mM NaCl, 5 mM Na_4_P_2_O_7_×10 H_2_O, 5 mM EDTA, 2% Triton X-100, adjusted to pH 7.3, plus protease inhibitor mix HP from Serva, www.serva.de) were normalized to protein concentrations with a detergent compatible protein assay (Bio-rad, www.biorad.com). Ten microgram protein were boiled for 5 min with 5× SDS sample buffer and subjected to SDS-PAGE gel electrophoresis using a 4% stacking gel and 10% separating gel. Proteins in the gel were electrophoretically transferred to nitrocellulose membranes, followed by immunoblotting with an antibody to PKC zeta (1∶1500; Santa Cruz Biotechnology) diluted in TBST with 5% milk. Bound antibody was visualized with horseradish peroxidase-conjugated secondary antibody (1∶2000, Santa Cruz Biotechnology) and enhanced chemiluminescence. Antibodies to glyceraldehyde-3-phosphate dehydrogenase (GAPDH, 1∶2500; www.abcam.com) were used as loading controls and were incubated along with the primary antibodies to PKC zeta.

### Data analysis

Unless indicated otherwise, values are given as means ± S.E.M. of N determinations. If one-way- or two-way (dependent on the experiment) repeated measures-corrected ANOVA yielded a p<0.05, groups were compared with Bonferroni post-hoc test.

Statistical tests were performed with SPSS® (www.spss.com).

## Results

**Figure 2:**
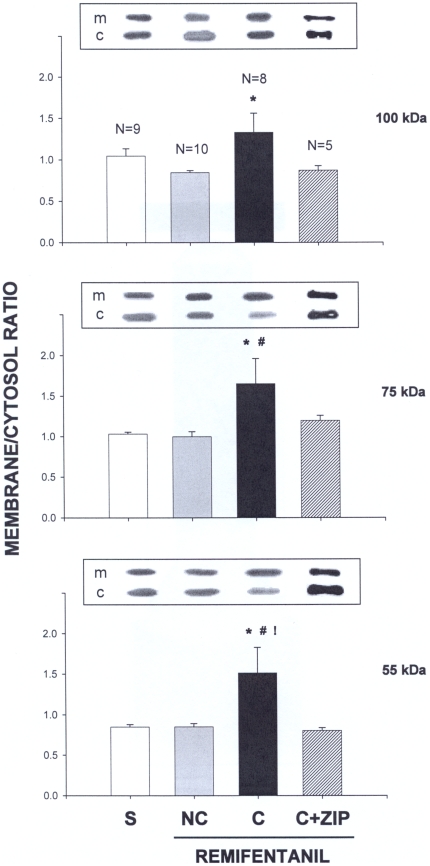
Acquisition and expression of conditioned remifentanil approach is paralleled by an activaction of PKC/Mzeta in the AcbC. The animals, the behavior of which is shown in Fig. 1, were sacrificed immediately after run #5 (see Fig. 1B), brain tissue lysates were obtained from the nucleus accumbens core (AcbC) and subcellular fractions were separated by ultracentrifugation. Ten micrograms of membrane- (m) or cytosolic (c) fraction were separated by SDS-PAGE, transferred to nitrocellulose membranes, and incubated with antibody (1∶1500) against PKCzeta (top panel, 100 kDa subunit band; middle panel; 75 kDa subunit band). This antibody also detected PKMzeta (bottom panel; 55 kDa band). Bound antibody was visualized with horseradish peroxidase-conjugated secondary antibody (1∶2000, Santa Cruz Biotechnology) and enhanced chemiluminescence. GAPDH immunoreactivity was used as a loading control. Activation of PKCzeta and PKMzeta is reflected by the membrane/cytosol ratio (abscissae; means ± SEM). S, saline (N = 9), NC, noncontingent remifentanil (N = 10), C, contingent remifentanil (N = 8), C+ZIP, contingent remifentanil followed by ZIP inhibition (N = 5). ANOVAs and Bonferroni post-hoc tests for each subunit gave the following results: 100 kDa subunit, F(3,28) = 3.001; p<0.05,* p<0.05 for C vs NC; 75 kDa subunit, F(3,28) = 3.698; p<0.023 * # p<0,05 for C vs NC and C vs S; and 55 kDa subunit, F(3,28) = 4,383; p<0.012; p<0.05 for C vs C+ZIP.

Experiment 1 (Figs. 1 and 2) tested the effects of acute PKC/Mzeta inhibition by ZIP which was administered locally into the AcbC after runway behavior was acquired during the first 4 response-i.v. remifentanil-pairings. Thus, Experiment 1 most likely assessed the effects of ZIP on the retrieval of memory regarding i.v. remifentanil-associated contextual conditioned stimuli (CS) and of the behavior previously associated with these contextual CSs. Acute ZIP administration increased the latency to leave the start area of the runway apparatus by about 25% (Fig. 1B1, assuming the latency difference between remifentanil-conditioned behaviour and saline-conditioned behavior to be 100%). ZIP also decreased the time that the rat spent in the goal area after the i.v. injection of remifentanil by about 30% (Fig. 1B3). Of note, running speed through the 1-m alley that connected the start area with the goal area was not affected by any treatment (i.e., contingent i.v. saline vs remifentanil or contingent remifentanil in absence or presence of acute intra-AcbC ZIP).

The acquisition and expression of conditioned remifentanil approach in the runway (Fig. 1) was paralleled by an activation of PKCzeta and PKMzeta in the AcbC, as evidenced (Fig. 2) by a shift in the membrane/cytosol ratio of both the 100 kDa and 75 kDa subunits of PKCzeta and PKMzeta (55 kDa). This effect was dependent on the contingent administration of i.v. remifentanil and could be fully inhibited by intra-AcbC ZIP. Noncontingent remifentanil, i.e., i.v. remifentanil that the rat passively received within the confines of the runway had no effect on PKCzeta and PKMzeta activation.

**Figure 3:**
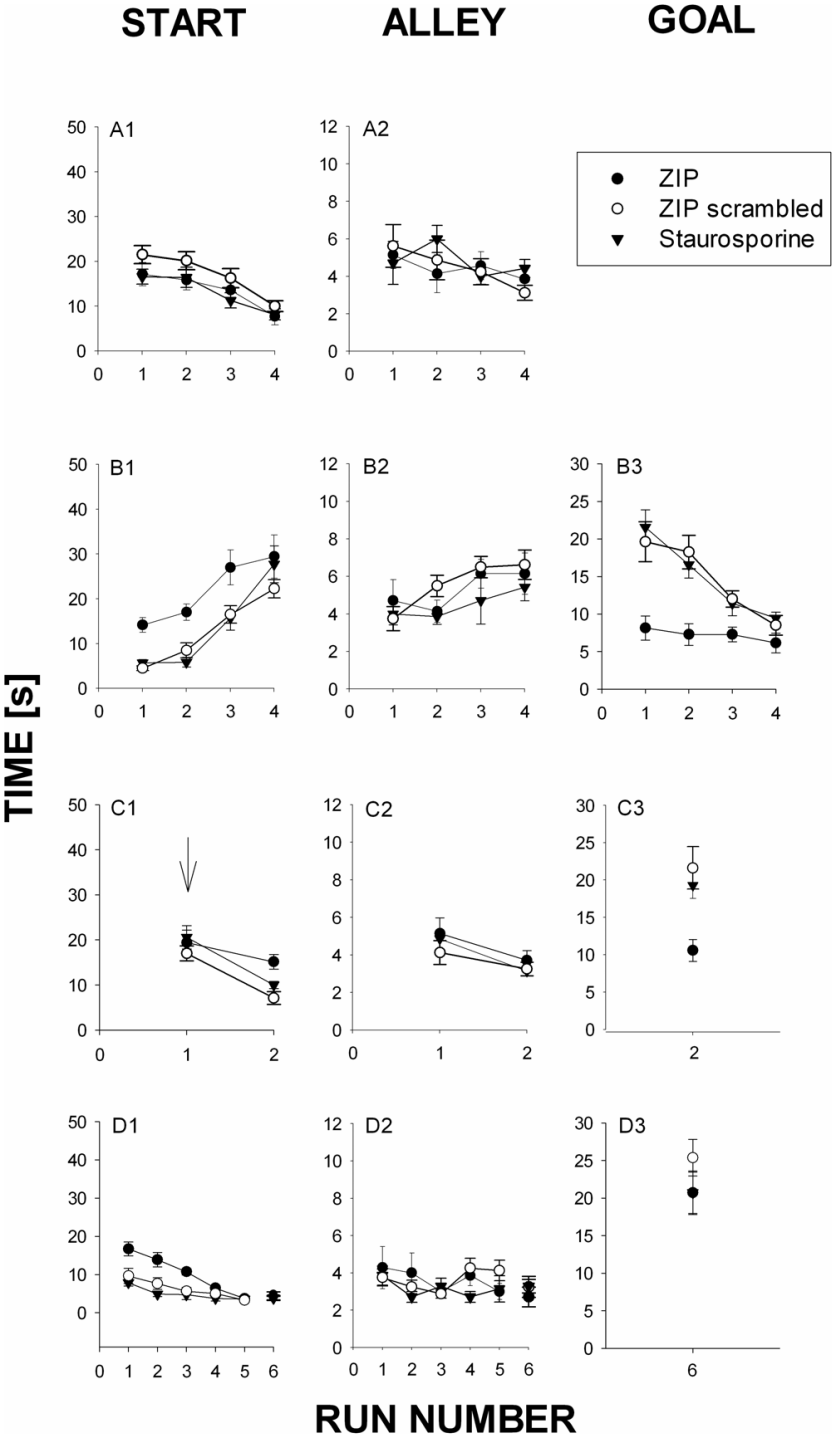
Inhibition of AcbC PKC/Mzeta blocks memory consolidation. Rats were trained to traverse a runway alley to obtain a remifentanil (RMF; 0.032 mg/kg iv) injection paired with a light stimulus (conditioned stimulus, CS; blinking at 2 Hz for 20 s) on day A (acquisiton; top row). The PKCzeta- and PKMzeta-pseudosubstrate inhibitor ZIP (N = 7), scrambled ZIP peptide (N = 8), or the nonselective PKC inhibitor staurosporine (N = 7) was administered locally into the nucleus accumbens core (0.5microliter of 1.5 mM each) 30 min before runs #1 and #4. On day B, only the light stimulus (CS) was presented when the animal had traversed the runway (all groups). One day later (day C), all animals received i.v. RMF upon traversing the alley in run #1. Traversing the runway in run #2 had no consequences. The next day (day D), all animals were retrained with RMF plus the light CS. No RMF was administered in run #5 in order to determine goal times. Column 1, start time; column 2; alley time; column 3, goal time (not determined on day A because of direct sedative effects of RMF). The statistical analysis yielded the following results: Panel B1: 2W-RM-ANOVA: Interaction: F(6,19) = 1,0327; run number: F(3,19) = 40,51 p<0,0001; Group F(2,19) = 9,771; p<0,01, posthoc (Bonferroni): ZIP vs ZIP-scrambled: runs 1,3 p<0,05. ZIPscramble vs Staurosporine: NS. ZIP vs staurosporine, runs 2,3 p<0.05. Panel B3: 1W-ANOVA Only for 1^st^ run. F(2,19) = 24.870; p<0.001. Posthoc/Bonferroni: ZIP vs ZIP-scramble: p<0.001; ZIP vs Stauro: p<0.001; ZIP-scramble vs Stauro: NS. Panel C1: 1W-ANOVA Only for 2^nd^ run. F(2,19) = 9.341; p<0.001, posthoc (Bonferroni). ZIP vs ZIP-scramble d; p<0.01. ZIP vs STAURO, p<0.05. staurosporine vs ZIP-scrambled. NS. Panel C3: 1W-ANOVA: F(2,19) = 6.995; p<0.01. Posthoc (Bonferroni) ZIP vs ZIP-scrambled p<0.01. ZIP–scrambled vs staurosporine, NS. ZIP vs staurosporine, p<0.05. Panel D1: 2W-RM-ANOVA. Interaction: F(8,19) = 3,106 p<0.01; Run Number F(4,19) = 22,17 p<0.0001. Group: F(2,19) = 22.51 p<0.0001. Posthoc (Bonferroni): ZIP vs ZIP-scrambled: runs 1,2 (p<0.01) and 3 (p<0.05). ZIP-scrambled vs staurosporine. NS. ZIP vs staurosporine runs 1,2,3 p<0.01. Panel D3: 1-W-ANOVA: F(2,19) = 1.044. NS.

Experiment 2 shows that PKCzeta and PKMzeta inhibition by ZIP was able to block memory consolidation while having no effect on memory acquisition. Although ZIP was administered intra-AcbC twice during the memory acquisition phase, i.e., before runs #1 and #4 of the first experimental day (Fig. 3A1), i.e., no effect was seen. Please note that such a ZIP administration pattern most plausibly resulted in appreciable intra-AcbC ZIP levels at the end of the experiment, too, thus affecting memory consolidation as well. Fig. 3B1 shows that on the next day, i.e., upon retesting the rats in the runway, ZIP which had been administered during the end of the previous day's experiment considerably increased start area latency before run #1, clearly indicating that ZIP had inhibited consolidation of the remifentanil-associated memory the day before. In contrast, scrambled ZIP or staurosporine, an effective inhibitor of c/nPKC-, CaMKII-, and PKA kinases that does not affect PKCzeta/PKMzeta activity [26], had no effect. ZIP also inhibited the increase in goal time engendered by i.v. remifentanil (Fig. 3B3). Again, scrambled ZIP or staurosporine failed to affect goal time. If a drug reminder cue was administered the next day (Fig. 3C), conditioned remifentanil approach was fully re-established, indicating that the drug cue was able to override any additional effect on memory reconsolidation ZIP may have exerted on the previous day. The inhibition of memory consolidation by ZIP was fully reversible, as the retraining session performed two days later shows (Fig. 3D). As in Experiment 1, alley running speed was not affected by any of the treatments.

**Figure 4:**
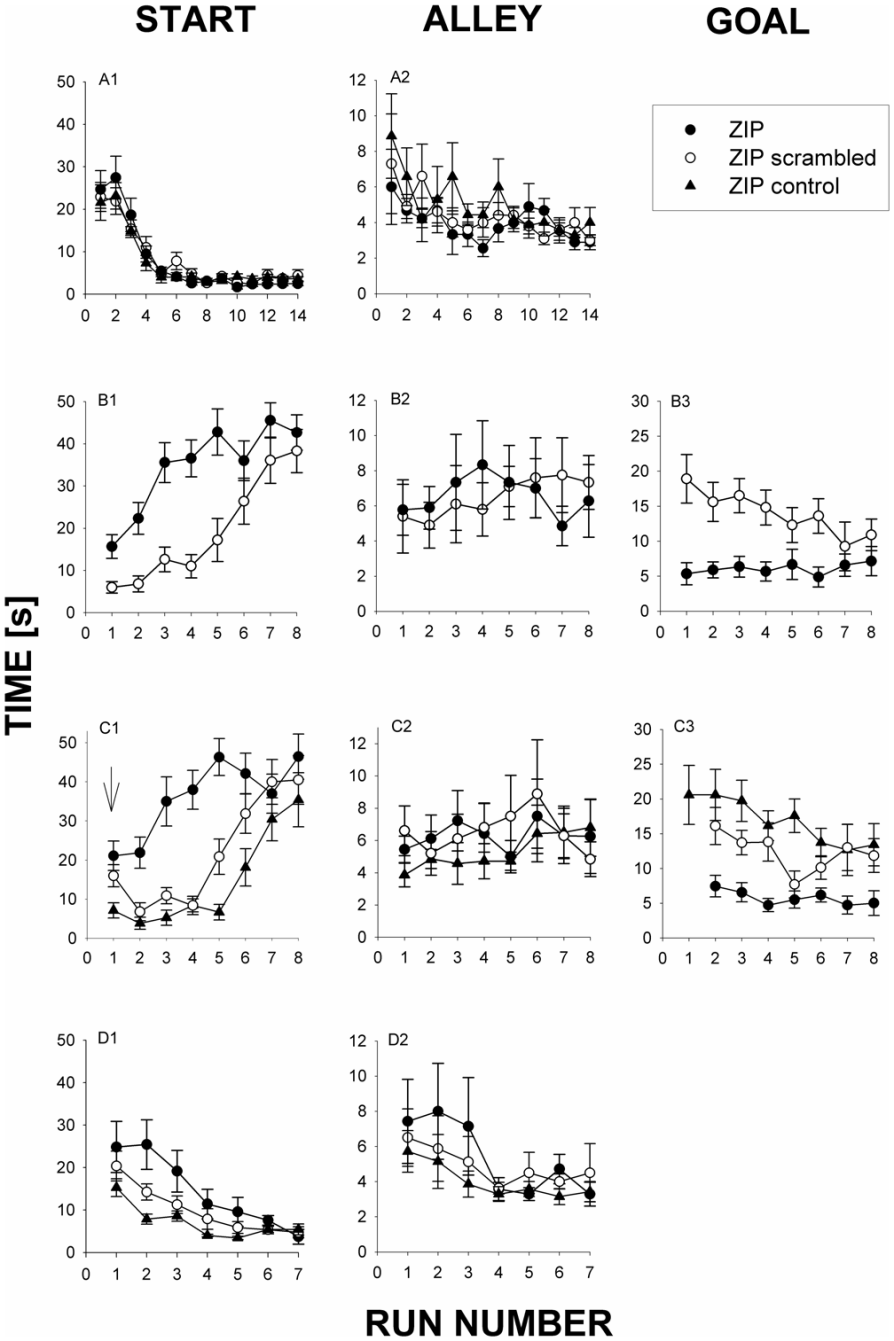
Inhibition of AcbC PKC/Mzeta inhibits memory reconsolidation. Rats were trained to traverse a runway alley to obtain a remifentanil (RMF; 0.032 mg/kg iv) injection paired with a conditioned light stimulus (conditioned stimulus, CS) on day A (top row). On day B (second row), animals received either ZIP (0.5microliter of 1.5 mM; N = 9) or scrambled ZIP peptide (N = 10) within the confines of the runway (30 min prior to starting run #1) and were presented only with the CS upon traversing the runway. The ZIPcontrol group (N = 7) received ZIP in their home cage without being tested in the runway. Two days later (C), the ZIP- and the scrambled peptide groups received an injection of RMF (drug-induced reinstatement of responding) only after the first run of the day (arrow); ZIP-control animals did not receive the drug reminder; all rats were presented with the CS upon entering the goal area. The next day (D), all animals were retrained with RMF+CS. Column 1, start time; column 2; alley time; column 3, goal time (not determined on days A and D because of confounding direct sedative effects of RMF). Column 1, start time; column 2; alley time; column 3, goal time (determined in absence of RMF). Note that on days B and C, that for all animals that did not leave the start area, no alley times and goal times could not be determined. Consequently, the number of animals that contributed to the mean times shown here were, on day B, run #3, N = 10 in the scrambled peptide group, and N = 9 in the ZIP group; run #4, 10 scrambled, 9 ZIP; run #5, 10 scrambled, 6 ZIP; run #6, 10 scrambled, 8 ZIP; run #7, 7 scrambled, 8 ZIP; and run #8, 7 scrambled, 9 ZIP. On day C, the respective numbers were: run #3, 10 scrambled, 9 ZIP, 7 ZIP-control; run #4, 10 scrambled, 7 ZIP, 7 ZIP-control; run #5, 10 scrambled, 6 ZIP, 7 ZIP-control; run #6, 8 scrambled, 6 ZIP, 7 ZIP-control; run #7, 7 scrambled, 7 ZIP, 6 ZIP-control; and run #8, 6 scrambled, 4 ZIP, and 5 ZIP-control. Statistical analysis yielded the following results: Panel B1: t-student for run #1: t = 3,202 with 17 degrees of freedom. (P = 0,005). Panel B3: t-student for run #1: t = 3,423 with 17 degrees of freedom. (P = 0,003). Panel C1: 1W-ANOVA for run #2: (ranks yields the same significance) F(2,23) = 10.660 p<0.001 Posthoc (Bonferroni): ZIP vs ZIP-control p<0.001- ZIP vs ZIP-scrambled p<0.01. ZIP-scrambled vs ZIP control.NS. Panel C3, run #1: only ZIP vs ZIP-CONTROL yielded significant in post-hoc comparison.

The effects of PKCzeta and PKMzeta inhibition on memory reconsolidation were tested in Experiment 3 (Fig. 4): After conditioned remifentanil approach had been acquired (Fig. 4A), animals were exposed to ZIP during a session (Fig. 4B) in which conditioned remifentanil approach was elicited by the light CS alone, plausibly leading to relevant ZIP levels during the reconsolidation phase after that session (Fig. 4B). If the rats were tested with the remifentanil reminder cue one day later (Fig. 4C), start latency (Fig. 4C1) was increased and goal time (Fig. 4C3) was decreased, strongly suggesting that the reconsolidation of these two components of the overall conditioned remifentanil approach behavior was inhibited. In contrast, if ZIP was administered noncontingently in the rats' home cages (ZIPcontrol) without the animals being exposed to the light CS, these behaviors were not changed. Interestingly, although scrambled ZIP did not affect behavior on the first run of that day (Figs. 4C1 and 4C3) – indicating that it did not affect reconsolidation, the treatment with scrambled ZIP speeded up extinction during the remifentanil reminder cue session as compared to the group that did receive ZIP in their home cage without undergoing the light CS reminder cue session. This plausibly indicates that the light CS reminder cue session constituted an extinction trial the effect of which, however, was first overridden by the drug reminder cue (run #1) but the extinction effect of which managed to emerge during the (unreinforced) subsequent runs of this session (Fig. 4C). As in all previous experiments, alley running speed was not affected by any of the treatments. All effects were reversible as the rats' behavior in the subsequent retraining session (Fig. 4D) shows.

## Discussion

Our findings indicate that local intra-AcbC activation of PKCzeta and PKMzeta but not PKC is necessary for the retrieval, consolidation and reconsolidation of drug-associated memories necessary for conditioned drug approach as determined in a rat runway paradigm [8], [9], confirming previous findings obtained in a lever-press-based self-administration paradigm [12]. The results of the present study strongly suggest PKCzeta and PKMzeta as a downstream target of the AcbC acetylcholine signal necessary for the acquisition of drug approach and, thus, conditioning of drug-associated contextual stimuli, an effect that seems to be preferential for drug- vs food reinforcers [9], but see [10], [11]. Using a very broad methodological approach including optogenetic inhibition, Deisseroth and co-workers [27] very plausibly identified cholinergic interneurons in the AcbC as the originator of the microdialysis signal observed by us previously [8], [9]. Optogenetic inhibition of these AcbC ACh interneurons prevented acquisition of cocaine CPP [27]. By generous extrapolation, one may assume that not only CPP behavior but “drug craving” may also be mediated by these AcbC ACh interneurons. As these AcbC ACh interneurons (1) comprise less than 1% of the Acb neuron population [28]and as (2) these AcbC ACh interneurons represent the AcbC's only known cholinergic input [29], they offer an ideal target for a therapeutic intervention.

The present findings contribute to the following anatomical differentiation regarding the different aspects of drug seeking (please see the discussion in [5]: While activation of the AcbC [4], [5] [4], [5](present study), and ACh interneuron activation in the AcbC (i.e., in the brain region medial of the anterior commissure) in particular [27], is necessary for establishing and maintaining drug-associated (contextual?) memories relevant for “maze” [17] behavior which is most likely strongly dependent on contextual cues (i.e., CPP- or runway behavior), operant conditioning, i.e., acquisition of a new operant response, depends on the activation of the BLA [5], [6], [7] but not the AcbC [5]. Accordingly, in our hands, local intra-AcbC PKCzeta and PKMzeta inhibition affected the latency to commit to traversing the runway and the time spent in the i.v. remifentanil injection-associated goal area (arguably a measure similar to the “time spent in the drug-associated compartment” as observed in the CPP paradigm), while not affecting the actual running speed (possibly the most “operant” component of the runway behavior).

Our findings also indicate that while a drug reminder (drug priming, see data of run #1 in figs. 4C1 and 4C3) overrides previous CS-based extinction training (Fig. 4B), subsequent extinction of drug primining-induced drug approach is facilitated by previous CS-based extinction training. Extrapolating to the human situation, our data indicate that while a drug-induced lapse may override previous therapeutic endeavors, these therapeutic interventions may still shorten the duration of drug-associated behavior (i.e., may prevent a full relapse), if extinction procedures are consequently applied. In other words, our findings suggest that, in the human situation, even post-lapse therapy may still be effective.

It has been argued that reconsolidation may be seen as accelerated extinction (p.76 of [3]. We have no indication that ZIP changed the speed of extinction (Figs. 3B1, Figs. 4B1).

Interestingly, acquisition of drug-conditioned place preference (CPP) could be blocked by intracerebroventricular injection of calphostin C (mouse morphine CPP [30]) or intra-Acb injection of NPC15437 (rat amphetamine CPP [31]). Neither of these two PKC inhibitors affects atypical PKCs [32]. It thus seems that activation of PKCzeta/PKMzeta is not necessary for the acquisition of drug-related memories whereas activation of other kinases is.

Although many addiction researchers would agree [23] that the rat runway paradigm constitutes a bona fide operant procedure (with traversing the runway alley as the operant), the most differentiating behavioral analysts [17] emphasize that the rat runway paradigm, like the conditioned place preference (CPP) procedure and other “maze” paradigms, is not able to distinguish between Pavlovian (i.e., respondent) approach (insensitive to changes in action-outcome contingencies and thus not truly goal-directed) and operant behavior (sensitive to changes in action-outcome contingencies). The rat runway paradigm, however, has proven sensitive to changes in action-outcome contingencies, as shown in the present study and in numerous previous ones [8], [9], [21], [22], [23].

In conclusion, the present results contribute to the following emerging picture: Drug-associated contextual stimuli acquire control over an individual's behavior through the activation of a a very localized group of neurons, i.e., the AcbC cholinergic interneurons [27], “accumbens core” designating a brain region medial of and adjacent to the anterior commissure. This effect is most likely mediated through the activation of M1 muscarinic receptors [33] and the atypical protein kinase C isoforms PKCzeta and PKMzeta in medium spiny neurons which are located immediately medial of the anterior commissure (i.e., neurons of the accumbal “core”) and which project most abundantly to the lateral ventral pallidum [34]. By extrapolation, these events may be part of the phenomenon sometimes reported by humans as “drug craving” [35]. These findings, based on a number of converging results obtained by several independent research groups using very different experimental approaches, offer hope for the targeted treatment of one of the most important determinants of drug lapse and relapse [36].
